# Supplementary material for: Clinical courses, impact and prognostic indicators for a persistent course of low back pain: Results from a population-based cohort study
Source: PLoS One. 2023 Mar 17;18(3):e0265104. doi: 10.1371/journal.pone.0265104 (PMC10022778; doi:10.1371/journal.pone.0265104)
Supplement: Attachment — Submitted filename: Peer review_PLoS ONE.docx [file pone.0265104.s002.docx]

I would like to thank the editor for giving me the chance to review this paper. The paper reports a large prospective study on the impact of persistent LBP on disability and health related quality of life as well as on predictors of long term LBP persistence. The paper reported a study conducted on an important and neglected health condition. Nevertheless, the manuscript has critical editing, organization, and structuring issues. There are lots of language problems as well as conceptual and methodological flaws throughout the paper that needs to be addressed before considering it for publication.

Below are my specific comments, concerns and suggestions in the different sections of the paper.

**Abstract**

1. The short title is not consistent with the main title. The main title indicates that the focus of the study is to find out the factors which predict long term LBP persistence, whereas, the short title is stated in such a way that the focus of the study is to determine the course and prognosis of low back pain.
2. The main title does not capture the study objective: “to assess the course of chronic LBP (CLBP) over 5 years in a large population-based study, its cumulative impact on disability and health-related quality of life (HRQoL) and the indicators for persistent CLBP course.”
3. It seems that the study is intended to achieve three specific objectives: i) determine the course of CLBP ii) investigate the cumulative impact of CLPB on disability and health related quality of life and iii) find out the indicators for persistent CLBP course. However, the title stated in such a way that the aim of the study is to find out the important factors which predict long term low back pain persistence. Hence, both the main title and the short title need medication in line with the objectives of the study.
4. It is not clear how the participants were selected. The author said that a representative sample of 10, 661 adults were randomly selected from the dwelling population of EpiDoC. But, how the study participants were selected?
5. Page 2, lines 35-36: “A General Linear Model was used to compare mean differences between and within groups.” What are the groups? Repeated measures ANOVA cannot be used to compare differences between groups.
6. The author concluded that “In the long-term, CLBP patients have distinct clinical courses.” What does this mean? No data were presented about clinical course. It seems that data are not presented for all of the three specific objectives the author claims to achieve. For instance, not data were presented on the course of CLBP.

**Background**

1. The background section is clear, well organized and provides context to the study. It also clearly shows the research gap. However, the aim of the study presented at the end of the background section (Page 6, lines 98-101) is not consistent with the data presented as well as the title of the study. For instance, no one can find data on the “presence of different LBP course patterns.”
2. The background section does not present synthesis of the existing evidence and research gaps related to the impact of CLBP on disability and HRQL and factors that predict CLBP persistence. What previous studies say with regard to the above research questions and what are the research gaps?

**Methods**

1. The author indicated that “The detailed procedures for the study design and sample selection and recruitment are published elsewhere” (Page 6, lines 110-111). This is Ok but there is need to briefly describe the study design and sample selection procedure as this paper has to be standalone. Need also to have “study design” and “sample selection” sub-sections.
2. The author needs to describe in detail the outcome measures of the Health Assessment Questionnaire (HAQ) and the EQ-5D-3L (number of items, content of items, psychometric properties and their appropriateness to the Portuguese situation). There is also need to provide more information about the HADS scale which was used to measure depression and anxiety.
3. It is not clear why disability and HRQL are considered as symptom-related indicators? Page 8, line 153.
4. The statement “Secondly, and as different CLBP courses were expected, descriptive statistics were planned for different CLBP groups” (Page 9, lines 167-168) is not clear at all.
5. Page 9, lines 170-171: “Differences between groups on disability and HRQoL, over time, were examined using repeated measures analysis of variance.” What are these groups? Repeated measures ANOVA assumes that the outcome variable (i. e. disability and HRQL in this case) needs to be normality distributed. Not clear whether or not this was checked.
6. What statistical method was used to analyze data to address one of the objectives of the study (i. e. to investigate the presence of different LBP course patterns across 5 years?

**Results**

1. Page 10, lines 195-196: “At baseline, no statistically significant differences in socio-demographic and clinical characteristics were found between responders and non-responders” Who are the responders and who are the non-responders at baseline? Do you mean those who had LBP and those who had not?
2. The age for the total sample is not reported (in Table 1). Why?
3. While the frequency for each category of educational level is reported, the percentage in brackets is not reported. Why?
4. Page 12, lines 233-235: “At any time-point there were statistically significant differences on disability (*p*<0.005) and HRQoL (EQ-5D-3L index, *p*<0.005), between groups.” It is possible to know this with a simple look at the descriptive data. However, it is not clear what statistical method was used to test this. Repeated measures ANOVA can only test within group differences, but not between group differences.
5. The statement “HRQoL statistically significantly decreased (*p*<0.001) and disability significantly increased (*p*<0.001) in the persistent group” (Page 13, lines 238-239) does not seem to be correct as the data on Table 2 doesn’t show the same.

**Discussion**

1. Overall, the discussion section is not well written and the major findings of the study are not properly interpreted. What the findings mean to the context where the study was conducted, and to specific nature of the sample? Findings are also not well related to previous literature, theory and treatment guidelines.
2. The implications of the findings to clinical practice and policy making are not described. Future directions to research in the area also are not indicated.
